# Arts-Based Programs for Older Adults for Their Cognition, Identity, Communication, Dignity, and More

**DOI:** 10.1093/geroni/igaa057.3057

**Published:** 2020-12-16

**Authors:** Kyong Hee Chee, Seoyoun Kim

## Abstract

This symposium takes an interdisciplinary perspective in order to address psychological and behavioral benefits of various arts-based programs for older adults including persons living with dementia. Presenters in this symposium specialize in diverse disciplines including psychology, social work, cognitive neuroscience, education, and sociology. Using novel approaches and various research methods, the presenters will speak to the specific outcomes of arts-based interventions. The positive outcomes include: 1) improved cognitive health rediscovered identities among cognitively normal older adults; and 2) better communication and reduced agitation for persons with dementia. The first two presentations concern cognitively intact participants. Brown will present a mixed-methods study that examined cognitive benefits and differential experiences of 11 older adults who participated in a 12-week, arts-based intervention. Chow will identify the themes in the ‘Tree of Life’ drawings of 144 Hong Kong Chinese older adults, who re-authored their sense of self transcending life challenges. Next, Mohan will discuss results from an analysis of communication exchanges among 6 older participants in a 6-week, creative group storytelling program (TimeSlips) offered in a memory care community. Halpin-Healy will explain the research-based practices used in museum programming (Arts & Minds) for persons with dementia and their care partners. She will summarize the assessments of the programs that have served approximately 500 participants over a decade. As a discussant, Kim will summarize common threads that lead to effective arts-based interventions for older adults regardless of their cognitive status. She will also highlight implications regarding the benefits of arts-based interventions in late life development.

